# Patterns of cognitive function in middle-aged and elderly Chinese adults—findings from the EMCOA study

**DOI:** 10.1186/s13195-018-0421-8

**Published:** 2018-09-15

**Authors:** Yu An, Lingli Feng, Xiaona Zhang, Ying Wang, Yushan Wang, Lingwei Tao, Yanhui Lu, Zhongsheng Qin, Rong Xiao

**Affiliations:** 1 School of Public Health, Capital Medical University, No.10 Xitoutiao, You An Men Wai, Beijing, 100069 China; 2 Peking university First Hospital, Beijing, China; 3 Linyi Mental Health Center, Linyi, Shandong China; 4 Jincheng People’s Hospital, Jincheng, Shanxi China

**Keywords:** Cognitive pattern, Gender-specific, Global and domain-specific, Normative data, Cross-sectional, Middle-aged and elderly

## Abstract

**Background:**

The principal aim of this study was to demonstrate the gender-specific cognitive patterns among middle-aged and elderly Chinese adults, investigate the risk factors on global and domain-specific cognitive performance in men and women, respectively, and report demographically adjusted norms for cognitive tests.

**Methods:**

The Effects and Mechanism of Cholesterol and Oxysterol on Alzheimer’s disease (EMCOA) study enrolled 4573 participants aged 50–70 years in three Chinese cities. All participants underwent an extensive neuropsychological test battery. Composite scores for specific domains were derived from principal component analysis (PCA). Multivariate linear regression models were used to determine gender-specific risk factors and demographically adjusted normative data.

**Results:**

Three cognitive domains of verbal memory, attention/processing speed/executive function, and cognitive flexibility were extracted. A female advantage in verbal memory was observed regardless of age, whereas men tended to outperform women in global cognition and attention/processing speed/executive function. The effects of education on women were more substantial than men for general cognition and attention/processing speed/executive function. For all the cognitive tests, regression-based and demographically adjusted normative data were calculated.

**Conclusions:**

There is a need for gender-specific intervention strategies for operationalizing cognitive impairment.

**Trial registration:**

EMCOA, ChiCTR-OOC-17011882. Retrospectively registered on 5 July 2017.

**Electronic supplementary material:**

The online version of this article (10.1186/s13195-018-0421-8) contains supplementary material, which is available to authorized users.

## Background

According to the World Alzheimer Report 2015 released by Alzheimer’s Disease International (ADI), 900 million people aged 60 years or above are now living worldwide, with this number expected to increase by 138–239% in middle-income countries such as China between 2015 and 2050 [1]. This is a noteworthy estimation given that normal aging is accompanied by deterioration across a spectrum of cognitive functions related to memory, attention, executive function, processing speed, and so on [2]. As a chronic and progressive neurodegenerative disorder that is strongly age-associated, dementia involves a severe loss of cognitive function beyond the normal aging process [3]. It can impede independent living and impose considerable personal, social, and economic burdens. Age-related cognitive impairment and the global impact of dementia has become a priority public health issue considering that the aging population constitutes a rapidly increasing proportion of the total population [4]. In the absence of an effective treatment, there is a responsibility for researchers to develop strategies to reduce the risk and slow the progression associated with mental aging.

Research on age-related cognitive impairment has shown that assessment of cognitive performance over the lifespan is a heterogeneous process [5]. On one hand, advanced age conveys positive influences on verbal abilities and production, and implicit and autobiographical memory due to growing knowledge and life experience. On the other hand, advanced age also conveys negative influences on processing speed, explicit memory, and verbal fluency due to age-related deterioration of the brain [6]. Diversity in cognitive performance and different rates of cognitive decline have been reported to be altered with regard to demographic characteristics, education, lifestyle, physical conditions, social engagement, and economic resources [7–9]. In fact, the influence of these sociodemographic characteristics on cognitive function is not homogeneous and they may interact with each other to yield distinctive patterns of cognitive performance. In particular, our previous studies have found that numerous cognitive scores were significantly different between men and women [10]. Lifestyle risk factors for mild cognitive impairment (MCI) are also gender-specific, in which smoking was only significant in men [11]. However, the gender-specific cognitive patterns and related risk factors are still under debate with respect to discrepant results across countries and are thus in need of further investigation. The elucidation of these different effects is crucial for understanding what determines healthy cognitive aging.

Including an estimated 218 million older people and 9.5 million people living with dementia, China has become a region with the most people living with dementia in 2015 [1]. Given this, many studies focused on older individuals in different stages of dementia, such as MCI [12–14]. Nevertheless, cognitive aging may begin in mid-life and has also been extensively investigated outside the context of dementia. Therefore, detection of cognitive decline in at-risk middle-aged and elderly groups has become a research priority [15]. Making firm identification and diagnosis between normal aging, MCI, and different subtypes of dementia requires the use of normative standards. Unbiased identification and diagnosis requires an individual’s cognitive performance to be compared to a normal sample from a comparable cognitively healthy population [16]. However, most commonly used neuropsychological tests only have norms for elderly populations aged 60 years or above. The norms for cognitive function are relatively under-researched among Chinese middle-aged and elderly adults owing to the lack of large-scale community-based studies. It can be problematic to draw clinical inferences from normative studies only for elderly populations aged 60 years or above.

A large-scale community-based study in China, the Effects and Mechanisms of Cholesterol and Oxysterol on Alzheimer’s disease (EMCOA) study, offers an opportunity to explore normal cognitive performance across the age spectrum of 50–70 years. This epidemiological investigation, begun in 2014, was primarily designed to prospectively determine the effects of dietary cholesterol and oxysterols on the incidence of Alzheimer’s disease (AD)/MCI in the middle-aged and elderly population. The present study emerged to investigate gender-specific cognitive patterns, explore risk factors for global or domain-specific cognitive performance in men and women, respectively, and to establish reliable normative information in Chinese middle-aged and elderly adults.

## Methods

### Setting

The present study was within the framework of the EMCOA study, an ongoing community-based cohort study of Chinese adults aged 50–70 years living in three Chinese cities of Beijing, Linyi, and Jincheng, and was registered on the Chinese Clinical Trial Registry as ChiCTR-OOC-17011882. The baseline examination took place between January 2014 and December 2015 and follow-up examinations take place every 2 years. The project was conducted by a synergistic collaboration among the Capital Medical University, Linyi Health Examination Center affiliated with Linyi People’s Hospital, Jincheng Health Examination Center affiliated with Jincheng People’s Hospital, and several community-based health centers affiliated with Beijing Chaoyang District Center for Disease Control and Prevention. Eligibility criteria for the EMCOA study included adults aged 50–70 years with no history of neuropsychiatric disorders or neoplastic diseases (malignant and benign tumor growths, e.g. head-neck tumors, metastatic lung, or upper digestive tumors) [17] and who simultaneously agreed to participate in the study. Exclusion criteria were as follows: 1) diagnosed with any neurodegenerative disease by neurologists (e.g., MCI or dementia); 2) suffering from cognitive impairment caused by depression, stroke, traumatic brain injury, or other severe organ dysfunction; 3) declined to participate in the study; 4) currently taking medication or dietary supplement to improve cognitive function; and 5) uncorrected visual or hearing impairment. The study protocols of the EMCOA study were reviewed and approved by the Ethics Committee of the Capital Medical University (2013SY35) and participants provided written informed consent.

### Study population

**Fig. 1:**
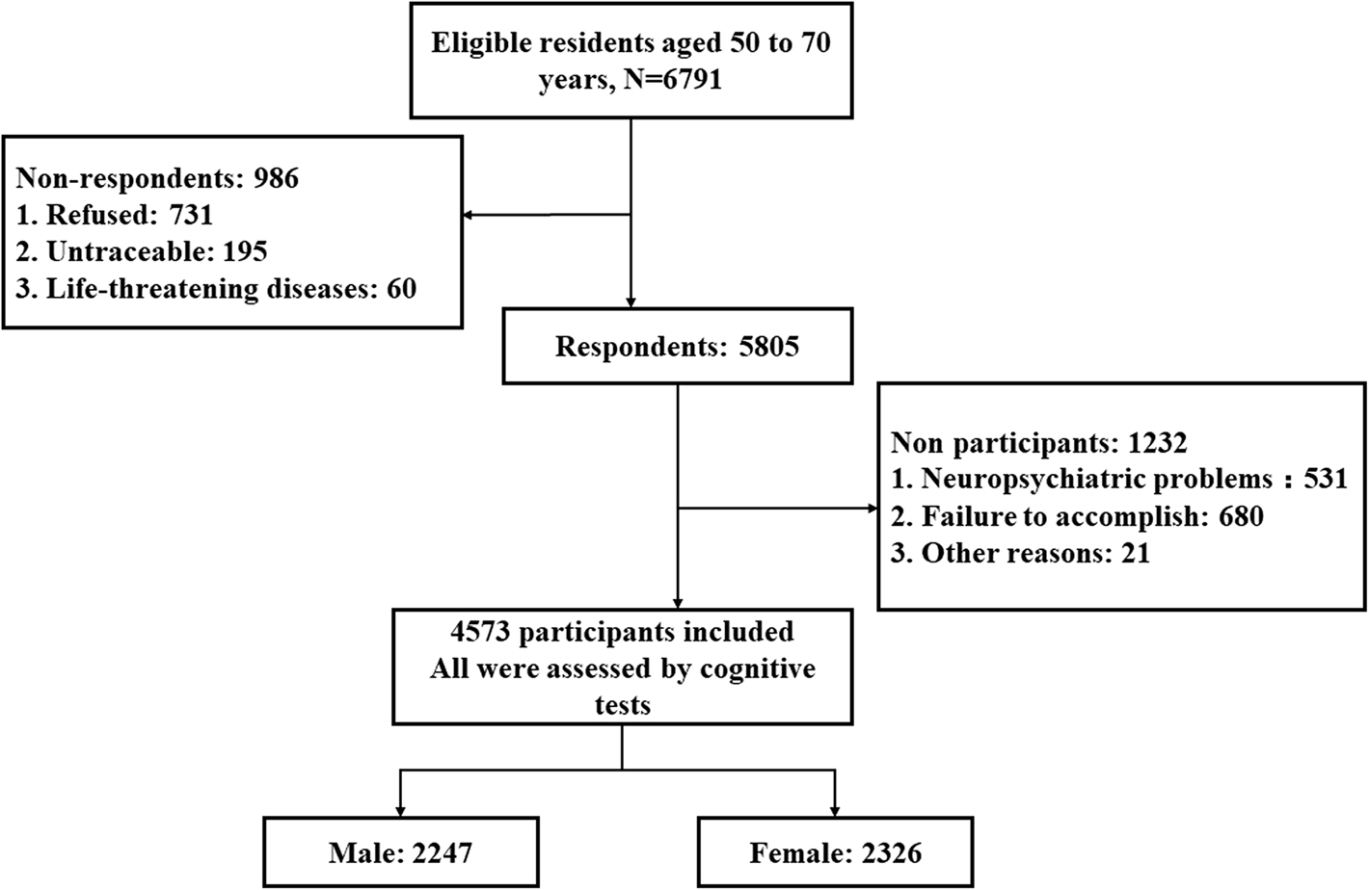
Study flow chart

The present analysis is based on the information obtained at the baseline examination. A total of 5805 individuals responded to the invitation and agreed to participate in this study. After checking the participants, 1232 participants were excluded for the following reasons: 531 due to neuropsychiatric problems (e.g., dementia, depression, or cerebral aneurysm), 680 due to the participant’s failure to complete the whole examination, and 21 due to other reasons. Finally, large cross-sectional data from 4573 middle-aged and elderly participants entered the study and were used for this analysis (Fig. 1). Of the 4573 participants, 2247 (49.1%) were men and 2326 (50.9%) were women.

### Cognitive test battery

Participants underwent neuropsychological evaluation in a private and quiet room carried out by technicians with formal training. A battery of well-validated Chinese version tests that possess high inter- and intra-rater reliability were administered to assess cognitive performance. Audio tape recordings of standardized testing procedures were reviewed across study sites to ensure consistency. We included the following cognitive tests: the Mini-Mental State Examination (MMSE) [18]; the Montreal Cognitive Assessment Test (MoCA) [19]; the Auditory Verbal Learning Test (AVLT) [20] using summarized scores of immediate recall (AVLT-IR), short recall (AVLT-SR), and long recall (AVLT-LR); the Symbol Digit Modalities Test (SDMT) [21]; the Wechsler Memory Scale Revised for China (WMS-RC) subtests Logical Memory Test—immediate recall (LMT-IR) [22], Digit Span Forwards (DSF), and Digit Span Backwards (DSB) [23]; the Trail Making Test (TMT) A and B [24]; and the Stroop Color-Word Test-Interference Trial (SCWT-IT) [25]. A detailed description of the procedure and modifications made to these measures can be found in Additional file 1: Supplementary methods and results.

### Covariates

At enrollment, a questionnaire on sociodemographics (gender, date of birth, years of formal education, employment, monthly household income, etc.), lifestyle (residence status, reading habits, physical activity, smoking, drinking, etc.), and clinical data (past and family medical history) was used to obtain information from the participants and/or their family member. Details of covariates are shown in Additional file 1.

### Data analysis

Principal component analysis (PCA) with varimax rotation was employed as a data-reduction technique to obtain composite scores for specific cognitive domains. The analysis of covariance was used to compare cognitive patterns between men and women. Sociodemographic characteristics, lifestyle, and medical variables, as well as cognitive performance between men and women, are reported as mean (standard deviation (SD)), median (interquartile range), or frequency (percentage). Reported *p* values refer to the Student *t* test, Mann Whitney *U* test, Kruskal-Wallis test, or chi-square test as appropriate. We used multivariate linear regression analysis for global and domain-specific cognitive performance as continuous outcomes. All models were adjusted for potential risk factors (sociodemographic characteristics, lifestyle, and medical variables) and stratified by gender. Heterogeneity of risk factors between men and women was assessed as gender × risk factor interactions which were included in overall models with the main effect terms. For interactions in multiple testing, an adjusted *p* value < 0.05, taking into account the false discovery rate (FDR) [26], was considered as statistically significant. The norms of these cognitive tests were also established and stratified according to variables that most associated with cognitive performance, and the details are shown in Additional file 1. All analyses were carried out using SPSS for Windows, version 23.0 (SPSS, Chicago, IL USA) and statistical significance was set at *p* < 0.05.

## Results

### Global and domain-specific cognitive performance

The means and SDs of all the cognitive tests are presented in Table 1. The PCA generated three principal components from 10 subtests with eigen values > 1 which accounted for 64.83% of the total initial variance in cognitive test performance (Table 2). The compound scores were calculated subsequently for: 1) verbal memory; 2) attention/processing speed/executive function; and 3) cognitive flexibility. The first component, primarily comprised of immediate, short, and long recall of AVLT, was interpreted to reflect verbal memory. The second component was interpreted to reflect attention/processing speed/executive function, with SDMT, LMT-IR, TMT A and B, DSF, and DSB contributing substantially. The third component was interpreted with SCWT-IT to reflect cognitive flexibility. The means and SDs of the composite scores of the three specific domains used in the analyses are presented in Table 3. All the cognitive tests had skewed distribution and the specific domains were symmetric.

**Table 1:** Cognitive tests of participants

Cognitive test	Total number	Mean	SD	Skew
MMSE	4494	28.11	2.137	−2.217
MoCA	4514	24.79	3.568	−1.242
AVLT-IR	4495	15.2209	4.98377	0.265
AVLT-SR	4483	5.23	2.522	0.040
AVLT-LR	4452	4.57	2.759	0.127
SDMT	4492	33.63	11.453	0.158
DSF	3923	7.72	1.448	−0.735
DSB	3920	4.03	1.292	−0.302
TMT-A	4486	69.75	27.295	1.029
TMT-B	4452	168.16	70.199	1.013
LMT-IR	4427	10.7408	5.10170	−0.067
SCWT-IT	4410	40.2502472	23.16964651	10.304

Skew > 0, positive skewed distribution; skew < 0, negative skewed distribution

*AVLT-IR* Auditory Verbal Learning Test—immediate recall, *AVLT-LR* Auditory Verbal Learning Test—long recall, *AVLT-SR* Auditory Verbal Learning Test—short recall, *DSB* digit span backwards, *DSF* digit span forwards, *MMSE* Mini-Mental State Examination, *MoCA* Montreal Cognitive Assessment, *SCWT-IT* Stroop Color-Word Test Interference Trial, *SDMT* Symbol Digit Modalities Test, *TMT* Trail Making Test, *LMT-IR* Logical Memory Test—immediate recall

**Table 2:** Principal components analysis for the cognitive subtests

Cognitive subtest	Components		
	Verbal memory	Attention/processing speed/executive function	Cognitive flexibility
AVLT-IR	**0.859348**	0.1786355	−0.02420138
AVLT-SR	**0.925347**	0.1656561	0.013152043
AVLT-LR	**0.919844**	0.1586545	0.007458428
SDMT	0.268641	**0.6900593**	0.107590145
DSF	0.056095	**0.5391598**	−0.48831854
DSB	0.256134	**0.5315925**	−0.36294081
TMT-A	0.09689	**0.7970535**	0.091785669
TMT-B	0.110346	**0.7881209**	0.142030366
LMT-IR	0.427479	**0.5010345**	−0.13808383
SCWT-IT	0.042578	0.2260081	**0.759748898**

Bold entries indicate measures with high loadings on each factor

*AVLT-IR* Auditory Verbal Learning Test—immediate recall, *AVLT-LR* Auditory Verbal Learning Test—long recall, *AVLT-SR* Auditory Verbal Learning Test—short recall, *DSB* digit span backwards, *DSF* digit span forwards, *SCWT-IT* Stroop Color-Word Test Interference Trial, *SDMT* Symbol Digit Modalities Test, *TMT* Trail Making Test, *LMT-IR* Logical Memory Test—immediate recall

**Table 3:** Characteristics of cognitive domains in participants

Cognitive domain	Total number	Mean	SD	Skew
Memory performance	3696	0.000	1.000	0.188
Attention/processing speed/executive function	3696	0.000	1.000	−0.624
Cognitive flexibility	3696	0.000	1.000	3.871

Each cognitive domain is the mean of the composite scores

Skew > 0, positive skewed distribution; skew < 0, negative skewed distribution

### Gender-specific cognitive patterns

Women scored better than men on verbal memory and cognitive flexibility, whereas men scored better on the MMSE, MoCA, and attention/processing speed/executive function (Table 4).

**Table 4:** Age group, education level, and cognitive performance between men and women

	Men (*n* = 2247)	Women (*n* = 2326)	*p* value
Age group (years), *n* (%)			0.086
50–54	550 (24.5%)	576 (24.8%)	
55–59	693 (30.8%)	767 (33.0%)	
60–64	671 (29.9%)	684 (29.4%)	
65–70	293 (13.0%)	251 (10.8%)	
Education level, *n* (%)			< 0.001**
Elementary school	218 (9.7%)	551 (23.7%)	
Junior middle school	666 (29.6%)	818 (35.2%)	
Senior middle school	678 (30.2%)	619 (26.6%)	
college and above	650 (28.9%)	293 (12.6%)	
Global cognitive function, mean (IQR)			
MMSE	29 (28, 30)	26 (24, 28)	< 0.001**
MoCA	28 (27, 30)	25 (22, 27)	< 0.001**
Domain-specific cognitive function, mean (IQR)			
Verbal memory	−0.10 (−0.73, 0.64)	0.03 (−0.68, 0.73)	0.003*
Attention/processing speed/executive function	0.29 (−0.38, 0.77)	−0.03 (−0.83, 0.59)	< 0.001**
Cognitive flexibility	−0.08 (−0.63, 0.39)	0.12 (−0.39, 0.67)	< 0.001**

Data shown as *n* (%) were compared between two groups using the chi-square test

Data with skewed distribution shown as median (interquartile range (IQR)) were compared between two groups using the Mann Whitney *U* test

*MMSE* Mini-Mental State Examination, *MoCA* Montreal Cognitive Assessment

∗*P* < 0.05; ∗∗*P* < 0.001

**Fig. 2:**
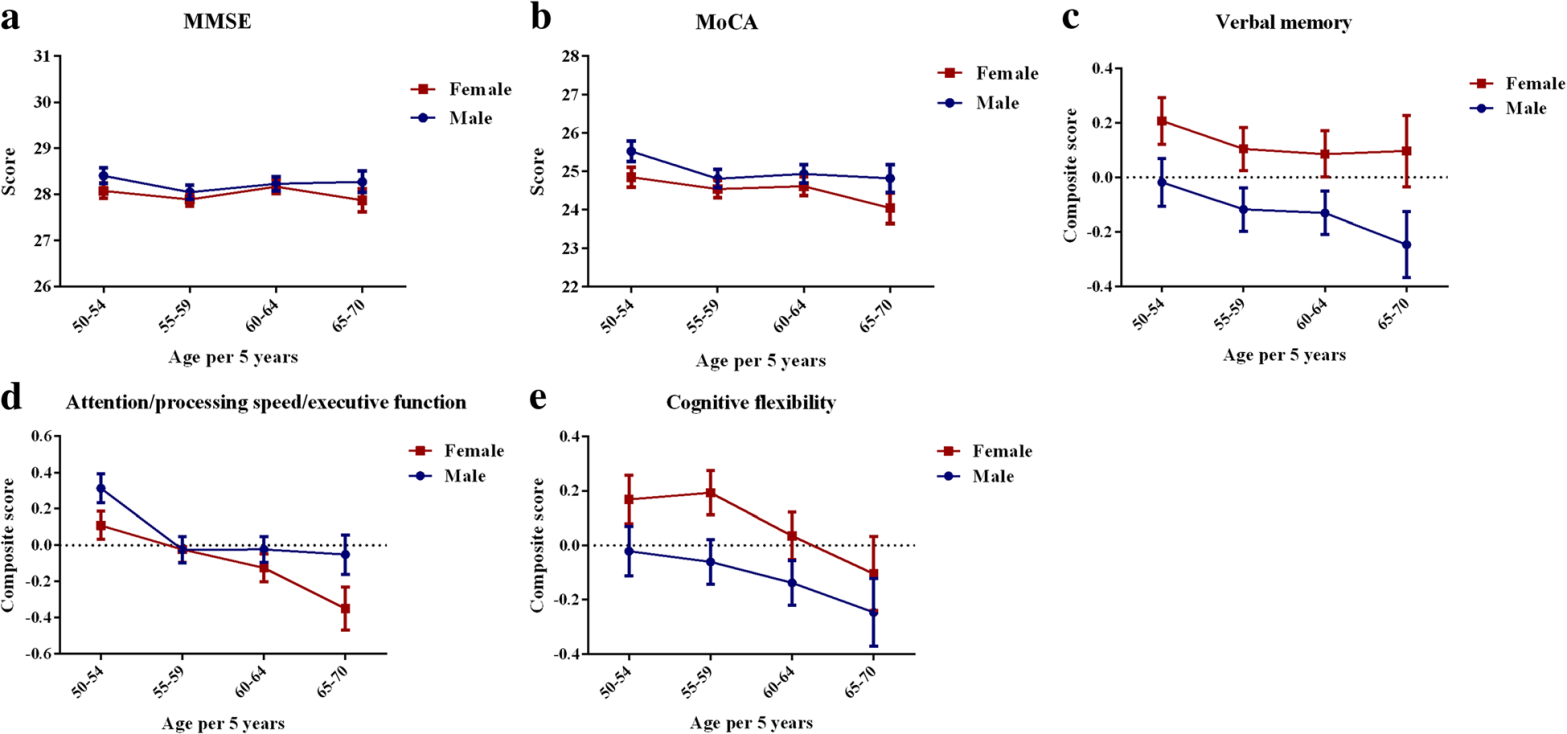
Gender-specific age effects on **a** Mini-Mental State Examination (MMSE), **b** Montreal Cognitive Assessment Test (MoCA), **c** verbal memory, **d** attention/processing speed/executive function, and **e** cognitive flexibility. The *x* axis represents age in 5-year groups and the *y* axis represents the scores. Error bars represent 95% confidence intervals. Estimates are adjusted for level of education

**Fig. 3:**
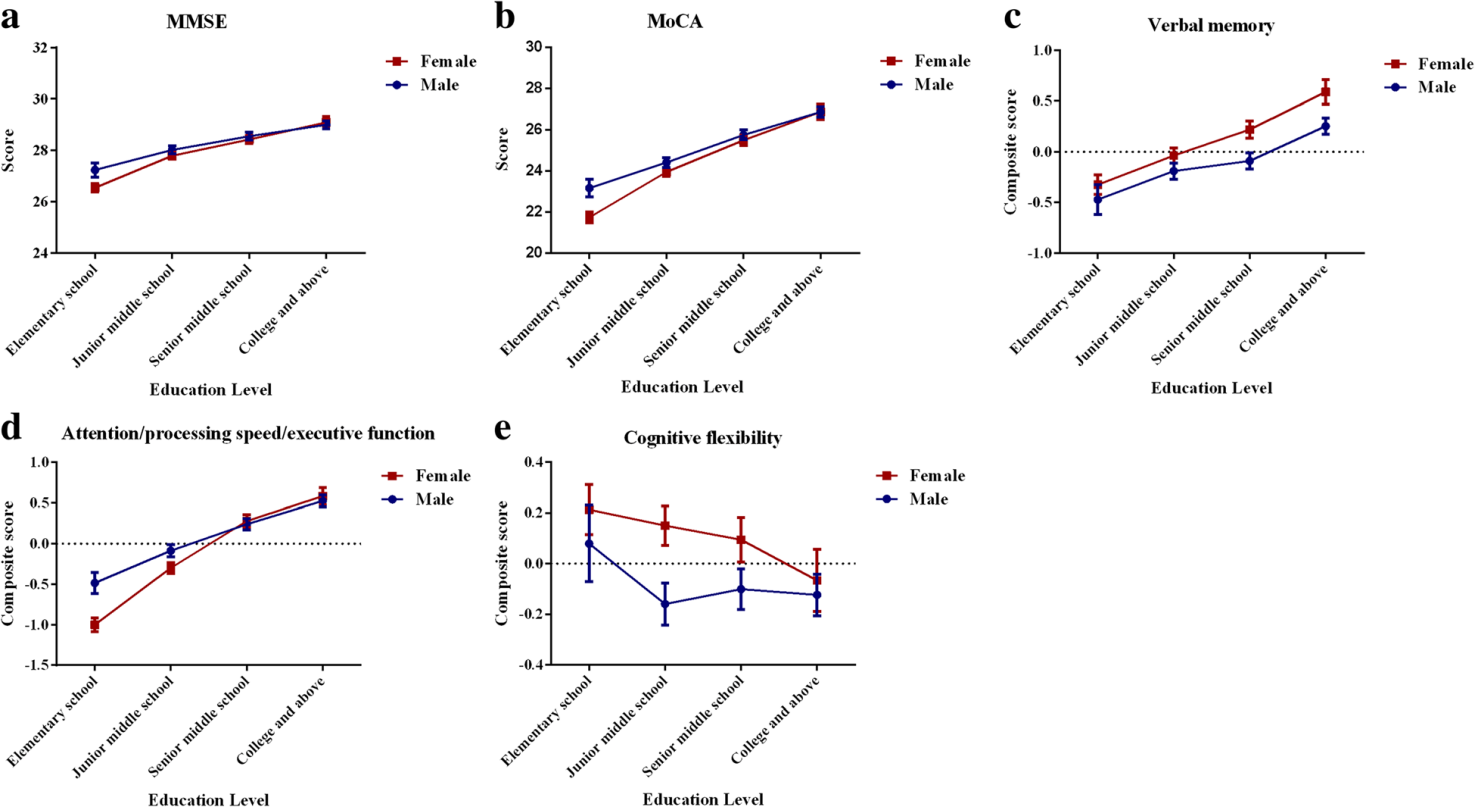
Gender-specific education effects on **a** Mini-Mental State Examination (MMSE), **b** Montreal Cognitive Assessment Test (MoCA), **c** verbal memory, **d** attention/processing speed/executive function, and **e** cognitive flexibility. The *x* axis represents education levels and the *y* axis represents the scores. Error bars represent 95% confidence intervals. Estimates are adjusted for age

The gender-specific cognitive patterns are presented in Figs. 2 and 3, which show mean levels and 95% confidence intervals (CIs) of cognitive performance stratified by age or education. On one hand, the female cognitive advantage across all ages was significant for verbal memory performance. Age was significantly associated with each cognitive measure in both men and women. On the other hand, a significant gender discrepancy existed for education level, and women tended to be less educated. In the elementary school educated group, women performed significantly worse than men in MMSE, MoCA, and attention/processing speed/executive function. However, this difference was eliminated in those with a higher education. In the senior middle school and college and above educated group, women performed the same as men in the aforementioned cognitive performance and even better than men for verbal memory. With respect to cognitive flexibility, women achieved significantly higher scores than men only for junior and senior middle school education.

### Gender-specific risk factors for cognitive performance

The comparison of sociodemographic characteristics, lifestyle, and medical variables between men and women are provided in Table 5. Compared with men, women included in our analysis were slightly younger (*p* = 0.04) and less likely to be engaged in white-collar work (*p* < 0.001). Women also reported lower education (*p* < 0.001) and income (*p* < 0.001). Meanwhile, a higher prevalence of being overweight and a lower prevalence of underweight body mass index (BMI) was observed in men compared with women (*p* < 0.001). With regard to lifestyle, men were more likely than women to be current smokers (*p* < 0.001) and to report current alcohol use and reading habits (*p* < 0.001). Differences in disease prevalence were such that men were more likely than women to report diabetes (*p* < 0.001), hypertension (*p* < 0.001), and coronary heart disease (*p* < 0.001), whereas women were more likely to have a family history of dementia (*p* = 0.039).

**Table 5:** General characteristics of the participants

	Men	Women	*p* value
Number, *n* (%)	2247 (49.1%)	2326 (50.9%)	–
Sociodemographic characteristics			
Age (years), median (IQR)	59 (55, 62)	58 (54, 62)	0.045*
Education (years), median (IQR)	12 (9, 15)	9 (9, 12)	< 0.001**
Occupation, *n* (%)			< 0.001**
Manual work	452 (20.1%)	723 (31.1%)	
White-collar work	921 (41.0%)	453 (19.5%)	
Monthly income, *n* (%)			< 0.001**
Low	607 (27.0%)	868 (37.3%)	
Medium	734 (32.7%)	722 (31.1%)	
High	906 (40.3%)	736 (31.6%)	
BMI (kg/m^2^), mean ± SD	25.38 ± 3.12	24.59 ± 3.16	< 0.001**
BMI group, *n* (%)			< 0.001**
Underweight	19 (0.8%)	27 (1.16%)	
Normal weight	992 (44.1%)	1268 (54.5%)	
Overweight	1018 (45.3%)	805 (34.6%)	
Obese	218 (9.7%)	226 (9.7%)	
Lifestyle, *n* (%)			
Solitude	42 (1.9%)	45 (1.9%)	0.871
Reading habits	1450 (64.5%)	1125 (48.4%)	< 0.001**
Physically active	1612 (71.7%)	1712 (73.6%)	0.158
Current smoker	973 (43.3%)	62 (2.7%)	< 0.001**
Current drinker	1123 (50.0%)	127 (5.5%)	< 0.001**
Medical history, *n* (%)			
Diabetes	372 (16.6%)	281 (12.1%)	< 0.001**
Hypertension	784 (34.9%)	664 (28.5%)	< 0.001**
Hyperlipidemia	476 (21.2%)	494 (21.2%)	0.964
Stroke	32 (1.4%)	26 (1.1%)	0.355
Coronary heart disease	203 (9.0%)	140 (6.0%)	< 0.001**
Family history of dementia	160 (7.1%)	204 (8.8%)	0.039*

Data shown as median (interquartile range (IQR)) were compared between two groups using the Mann Whitney *U* test

Data shown as mean ± standard deviation (SD) were compared between two groups using the Student *t* test

Data shown as *n* (%) were compared between two groups using the chi-square test

*BMI* body mass index

∗*P* < 0.05; ∗∗*P* < 0.001

We examined the gender-specific risk factors on cognitive performance using multivariate analysis (Table 6) and found that sociodemographic, lifestyle, and medical variables had different effects on cognitive performance in men and women. For sociodemographic characteristics, male global and domain-specific cognitive performance was positively associated with education, intellectual occupation, and higher monthly income, whereas it was negatively associated with age. Similarly, female cognitive performance was also positively associated with education and a white-collar occupation and negatively associated with age. Furthermore, being underweight and obesity also negatively impacted female verbal memory and attention/processing speed/executive function. For lifestyle, both male and female global cognitive performance and verbal memory benefited from reading habits. Meanwhile, solitude and smoking were negatively associated with male global cognitive score and verbal memory while being physically active had a positive influence on male attention/processing speed/executive function. For medical variables, diabetes and coronary heart disease were associated with lower verbal memory score in men, hypertension was associated with lower MoCA scores in women, and stroke was associated with a lower MMSE score in men and cognitive flexibility score in women. Significant differences between men and women were observed for an association of years of education with MMSE, MoCA, and attention/processing speed/executive function. The effects of increased education years on general cognition and attention/processing speed/executive function were significantly greater in women than men (*p* < 0.001 for interaction, and *p* < 0.05 after FDR adjustment).

**Table 6:** Gender-specific associations of sociodemographic characteristics, lifestyle, and medical history with cognitive performance

	MMSE		*p* ^*a*^	MoCA		*p*	Verbal memory		*p*	Attention/processing speed/executive function		*p*	Cognitive flexibility		*p*
	Men	Women		Men	Women		Men	Women		Men	Women		Men	Women	
Sociodemographic characteristics															
Age	−0.038	−0.031	0.446	−0.078*	−0.063	0.327	−0.054	−0.114*	0.187	−0.185**	−0.110*	0.923	−0.096*	−0.042	0.311
Education years	0.163**	0.308**	< 0.001^#^	0.225**	0.369**	< 0.001^#^	0.087	0.196**	0.226	0.253**	0.403**	< 0.001^#^	−0.08	−0.037	0.128
Occupation															
Manual work	Ref	Ref	Ref	Ref	Ref	Ref	Ref	Ref	Ref	Ref	Ref	Ref	Ref	Ref	Ref
White-collar work	0.01	0.067	0.069	0.110*	0.066	0.118	0.100*	0.081	0.954	0.104*	0.155**	0.006	−0.007	0.004	0.114
Monthly income															
Low	Ref	Ref	Ref	Ref	Ref	Ref	Ref	Ref	Ref	Ref	Ref	Ref	Ref	Ref	Ref
Medium	0.133*	0.069	0.656	0.083*	0.058	0.557	0.026	0.043	0.401	0.055	−0.004	0.995	0.107*	−0.008	0.933
High	0.179**	0.058	0.96	0.137*	0.056	0.888	0.133*	0.063	0.306	0.069	−0.023	0.589	0.157*	−0.064	0.059
Body mass index															
Healthy	Ref	Ref	Ref	Ref	Ref	Ref	Ref	Ref	Ref	Ref	Ref	Ref	Ref	Ref	Ref
Underweight	0.008	−0.003	0.939	−0.001	−0.004	0.807	−0.021	−0.101*	0.222	−0.012	0.034	0.198	0.002	0.09	0.133
Overweight	0.041	−0.036	0.426	0.042	−0.063	0.034	0.092	−0.015	0.316	−0.029	−0.049	0.778	0.021	0.02	0.295
Obesity	0.076	−0.039	0.111	0.073	0.011	0.93	0.085	−0.023	0.271	−0.038	−0.073*	0.243	−0.038	0.052	0.078
Lifestyle															
Solitude	−0.043	0.051	0.045	−0.095*	0.005	0.045	−0.013	0.028	0.415	0.007	0.007	0.929	−0.009	0.026	0.271
Reading habits	0.079*	0.097*	0.113	0.169**	0.185**	0.051	0.143**	0.123*	0.729	−0.002	0.056	0.022	0.031	−0.118	0.057
Physically active	0.015	−0.063	0.295	−0.046	−0.07	0.726	−0.038	−0.053	0.892	0.100*	0.046	0.917	0.029	−0.019	0.343
Current smoker	−0.074*	−0.023	0.984	−0.058*	−0.001	0.467	−0.102*	−0.001	0.2	−0.016	−0.002	0.894	−0.057	0.019	0.277
Current drinker	0.055	0.012	0.922	0.059	0.042	0.4	0.063	0.064	0.281	0.014	−0.001	0.996	0.015	−0.015	0.61
Medical history															
Diabetes	−0.009	−0.01	0.961	0.002	−0.002	0.967	−0.071*	−0.021	0.487	−0.008	−0.031	0.559	0.002	−0.037	0.548
Hypertension	−0.007	−0.047	0.343	−0.048	−0.074*	0.343	−0.036	−0.036	0.852	0.014	−0.048	0.08	−0.053	−0.002	0.245
Hyperlipidemia	−0.004	0.021	0.285	0.015	0.021	0.463	0.032	0.007	0.997	0.017	0.067	0.282	0.01	0.074	0.257
Stroke	−0.106*	−0.006	0.063	−0.035	0.001	0.475	−0.014	0.042	0.234	−0.002	−0.043	0.284	0.031	−0.100*	0.061
Coronary heart disease	−0.056	0.05	0.023	−0.053	0.03	0.081	−0.142**	−0.027	0.069	0.082	0.043	0.602	0.018	0.011	0.79
Family history of dementia	−0.041	0.013	0.207	−0.01	0.017	0.446	0.013	0.041	0.612	−0.026	0.022	0.309	−0.056	−0.056	0.989

Multivariate linear regression analysis with the enter method was performed on all variables and standardized regression coefficients are presented

*MMSE* Mini-Mental State Examination, *MoCA* Montreal Cognitive Assessment

^a^*p* for interactions

*P < 0.05, **P < 0.001; false discovery rate adjusted ^#^*P* < 0.05

### Development of normative data for 12 cognitive tests and related *z* score

**Fig. 4:**
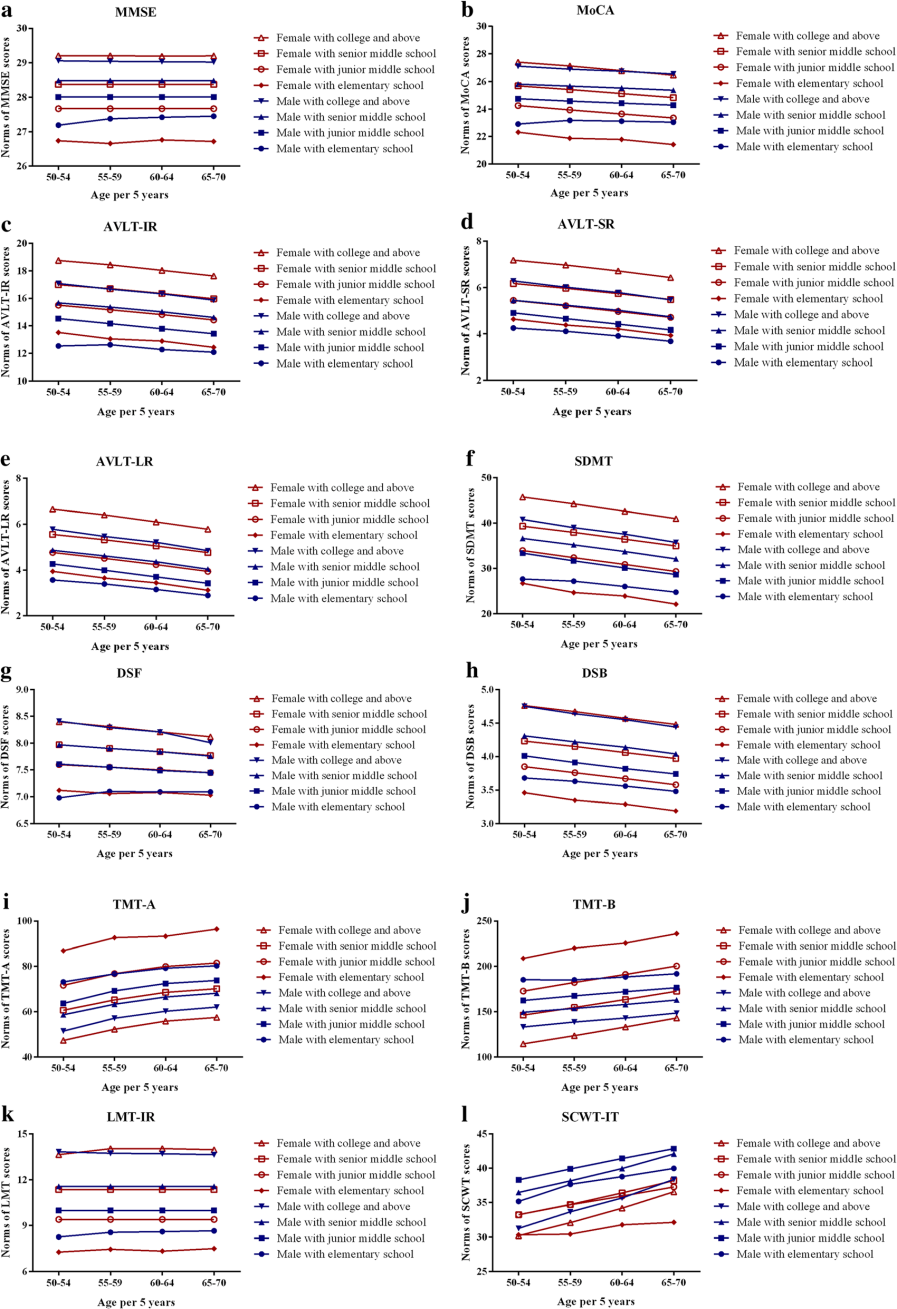
Gender-, age-, and education-adjusted norms of (**a**) Mini-Mental State Examination (MMSE), (**b**) Montreal Cognitive Assessment Test (MoCA), (**c**) Auditory Verbal Learning Test—immediate recall (AVLT-IR), and (**d**) Auditory Verbal Learning Test—short recall (AVLT-SR). Gender-, age-, and education-adjusted norms of (**e**) Auditory Verbal Learning Test—long recall (AVLT-LR), (**f**) Symbol Digit Modalities Test (SDMT), (**g**) Digit span forwards (DSF), and (**h**) Digit span backwards (DSB). Gender-, age-, and education-adjusted norms of (**i**) Trail Making Test (TMT)-A, (**j**) TMT-B, (**k**) Logical Memory Test—immediate recall (LMT-IR), and (**l**) Stroop Color-Word Test Interference Trial (SCWT-IT)

The predictive scores and normative data were developed based on three variables of age, gender, and education from multivariate regression models (Table 7). The equations are shown in Additional file 1 and the regression coefficients are presented in Table 8. Next, the predictive scores were used to generate demographically adjusted *z* scores which can be converted to a percentile that indicates the individual’s cognitive performance among peers of comparable age, gender, and education. The normative data of 12 cognitive tests were determined and stratified by age, gender, and education (Table 9, Fig. 4a-l). Furthermore, the reference cut-off values are also shown (Table 10) to define cognitive impairment.

**Table 7:** Proportion of variance accounted for cognitive performance in linear regression analyses for 12 cognitive tests

Cognitive tests	Model 1	Model 2	△ *R*^2^
MMSE	0.150	0.176	0.026
MoCA	0.255	0.314	0.059
AVLT-IR	0.124	0.161	0.037
AVLT-SR	0.118	0.16	0.042
AVLT-LR	0.118	0.168	0.050
SDMT	0.279	0.304	0.025
DSF	0.084	0.108	0.024
DSB	0.140	0.159	0.019
TMT-A	0.214	0.232	0.018
TMT-B	0.172	0.193	0.021
LMT-IR	0.201	0.242	0.041
SCWT-IT	0.016	0.018	0.002

The values represent the proportion of variance (*R*^2^) in the regression model

In model 1, the linear regression analysis was performed only on age, gender, and education

In model 2, the linear regression analysis was performed on all the sociodemographic, lifestyle, and medical variables

Both models used the enter method

*AVLT-IR* Auditory Verbal Learning Test—immediate recall, *AVLT-LR* Auditory Verbal Learning Test—long recall, *AVLT-SR* Auditory Verbal Learning Test—short recall, *DSB* digit span backwards, *DSF* digit span forwards, *MMSE* Mini-Mental State Examination, *MoCA* Montreal Cognitive Assessment, *SCWT-IT* Stroop Color-Word Test Interference Trial, *SDMT* Symbol Digit Modalities Test, *TMT* Trail Making Test, *LMT-IR* Logical Memory Test—immediate recall

**Table 8:** Regression coefficients of the normative data equations

Cognitive tests	Gender	Age	Education	Gender × age	Gender × education	Age × education	Age^2^	Education^2^	Constant
MMSE	−1.041	–	0.084	–	0.078	–	–	–	27.599
MoCA	0.127	–	0.233	−0.031	0.127	–	–	–	23.136
AVLT-IR	–	–	0.282	–	0.11	–	−0.001	–	12.727
AVLT-SR	–	–	–	–	0.06	–	−0.001	0.006	5.028
AVLT-LR	–	–	–	–	0.057	–	−0.001	0.007	4.505
SDMT	−5.477	−0.323	0.461	–	0.681	–	–	–	45.41
DSF	–	–	0.186	–	–	−0.001	–	–	6.502
DSB	−0.391	−0.019	–	–	0.026	–	–	0.004	4.864
TMT-A	24.601	5.309	–	–	−1.877	–	−0.039	–	−114.999
TMT-B	–	–	–	0.969	−4.493	–	–	–	152.416
LMT-IR	−1.751	–	–	–	0.129	–	–	0.019	9.071
SCWT-IT	−0.277	–	–	–	0.005	0.001	–	−0.004	3.686

The values represented unstandardized regression coefficient

*AVLT-IR* Auditory Verbal Learning Test—immediate recall, *AVLT-LR* Auditory Verbal Learning Test—long recall, *AVLT-SR* Auditory Verbal Learning Test—short recall, *DSB* digit span backwards, *DSF* digit span forwards, *MMSE* Mini-Mental State Examination, *MoCA* Montreal Cognitive Assessment, *SCWT-IT* Stroop Color-Word Test Interference Trial, *SDMT* Symbol Digit Modalities Test, *TMT* Trail Making Test, *LMT-IR* Logical Memory Test—immediate recall

**Table 9:** Regression-based normative data of cognitive performance stratified by age, gender, and education as appropriate

		Education level							
		Elementary school		Junior middle school		Senior middle school		College and above	
	Age	Male	Female	Male	Female	Male	Female	Male	Female
MMSE	50–54	27.19 ± 0.47	26.74 ± 0.51	28.01 ± 0.00	27.67 ± 0.00	28.49 ± 0.00	28.38 ± 0.00	29.06 ± 0.08	29.21 ± 0.12
	55–59	27.38 ± 0.35	26.66 ± 0.58	28.01 ± 0.00	27.67 ± 0.00	28.49 ± 0.00	28.38 ± 0.00	29.04 ± 0.08	29.21 ± 0.12
	60–64	27.42 ± 0.30	26.76 ± 0.48	28.01 ± 0.00	27.67 ± 0.00	28.49 ± 0.00	28.38 ± 0.00	29.03 ± 0.08	29.19 ± 0.12
	65–70	27.45 ± 0.26	26.72 ± 0.53	28.01 ± 0.00	27.67 ± 0.00	28.49 ± 0.00	28.38 ± 0.00	29.02 ± 0.07	29.20 ± 0.12
MoCA	50–54	22.91 ± 1.06	22.32 ± 1.04	24.75 ± 0.04	24.25 ± 0.08	25.81 ± 0.04	25.69 ± 0.08	27.10 ± 0.19	27.40 ± 0.25
	55–59	23.18 ± 0.77	21.88 ± 1.18	24.58 ± 0.04	23.94 ± 0.08	25.67 ± 0.04	25.42 ± 0.09	26.89 ± 0.18	27.12 ± 0.27
	60–64	23.12 ± 0.68	21.79 ± 1.00	24.43 ± 0.04	23.65 ± 0.09	25.53 ± 0.05	25.13 ± 0.09	26.74 ± 0.18	26.78 ± 0.24
	65–70	23.05 ± 0.58	21.42 ± 1.07	24.29 ± 0.05	23.35 ± 0.10	25.37 ± 0.05	24.84 ± 0.09	26.54 ± 0.17	26.47 ± 0.27
AVLT-IR	50–54	12.54 ± 1.15	13.52 ± 1.07	14.53 ± 0.09	15.50 ± 0.08	15.67 ± 0.08	16.99 ± 0.08	17.08 ± 0.23	18.75 ± 0.26
	55–59	12.63 ± 0.83	13.05 ± 1.21	14.16 ± 0.10	15.17 ± 0.10	15.36 ± 0.10	16.70 ± 0.10	16.67 ± 0.22	18.44 ± 0.29
	60–64	12.28 ± 0.75	12.90 ± 1.03	13.79 ± 0.11	14.82 ± 0.11	15.02 ± 0.11	16.35 ± 0.11	16.33 ± 0.22	18.04 ± 0.25
	65–70	12.09 ± 0.64	12.44 ± 1.10	13.43 ± 0.15	14.43 ± 0.13	14.61 ± 0.13	15.97 ± 0.12	15.87 ± 0.24	17.63 ± 0.28
AVLT-SR	50–54	4.25 ± 0.28	4.64 ± 0.33	4.91 ± 0.06	5.44 ± 0.06	5.45 ± 0.05	6.17 ± 0.05	6.28 ± 0.15	7.18 ± 0.16
	55–59	4.11 ± 0.21	4.38 ± 0.38	4.66 ± 0.07	5.21 ± 0.07	5.24 ± 0.07	5.97 ± 0.07	6.01 ± 0.14	6.97 ± 0.18
	60–64	3.91 ± 0.20	4.21 ± 0.33	4.42 ± 0.07	4.97 ± 0.07	5.02 ± 0.08	5.74 ± 0.07	5.78 ± 0.15	6.71 ± 0.15
	65–70	3.68 ± 0.19	3.93 ± 0.34	4.17 ± 0.10	4.71 ± 0.09	4.74 ± 0.09	5.48 ± 0.08	5.47 ± 0.16	6.43 ± 0.19
AVLT-LR	50–54	3.57 ± 0.30	3.94 ± 0.34	4.27 ± 0.07	4.77 ± 0.06	4.86 ± 0.06	5.55 ± 0.06	5.78 ± 0.16	6.65 ± 0.18
	55–59	3.39 ± 0.22	3.65 ± 0.38	3.99 ± 0.08	4.51 ± 0.08	4.62 ± 0.08	5.32 ± 0.08	5.47 ± 0.16	6.40 ± 0.20
	60–64	3.15 ± 0.21	3.44 ± 0.33	3.70 ± 0.09	4.24 ± 0.08	4.36 ± 0.09	5.05 ± 0.08	5.21 ± 0.16	6.10 ± 0.17
	65–70	2.89 ± 0.20	3.12 ± 0.35	3.42 ± 0.11	3.94 ± 0.10	4.04 ± 0.10	4.76 ± 0.10	4.85 ± 0.18	5.78 ± 0.21
SDMT	50–54	27.67 ± 3.34	26.70 ± 3.88	33.38 ± 0.46	33.95 ± 0.40	36.64 ± 0.39	39.35 ± 0.39	40.81 ± 0.76	45.76 ± 0.97
	55–59	27.18 ± 2.43	24.68 ± 4.40	31.68 ± 0.44	32.38 ± 0.44	35.20 ± 0.46	37.96 ± 0.45	39.02 ± 0.74	44.27 ± 1.09
	60–64	26.00 ± 2.22	23.91 ± 3.74	30.10 ± 0.46	30.88 ± 0.45	33.74 ± 0.47	36.46 ± 0.45	37.57 ± 0.73	42.62 ± 0.91
	65–70	24.76 ± 1.92	22.11 ± 4.00	28.66 ± 0.56	29.34 ± 0.50	32.12 ± 0.49	34.95 ± 0.48	35.76 ± 0.80	40.98 ± 1.09
DSF	50–54	6.98 ± 0.36	7.12 ± 0.26	7.61 ± 0.02	7.60 ± 0.01	7.97 ± 0.02	7.97 ± 0.02	8.41 ± 0.07	8.40 ± 0.06
	55–59	7.10 ± 0.25	7.06 ± 0.28	7.55 ± 0.01	7.55 ± 0.01	7.90 ± 0.02	7.90 ± 0.02	8.29 ± 0.06	8.31 ± 0.07
	60–64	7.09 ± 0.21	7.08 ± 0.22	7.49 ± 0.02	7.50 ± 0.02	7.84 ± 0.02	7.84 ± 0.02	8.21 ± 0.06	8.21 ± 0.06
	65–70	7.09 ± 0.17	7.03 ± 0.23	7.45 ± 0.02	7.45 ± 0.02	7.76 ± 0.02	7.77 ± 0.02	8.01 ± 0.06	8.12 ± 0.06
DSB	50–54	3.68 ± 0.14	3.46 ± 0.16	4.01 ± 0.03	3.85 ± 0.02	4.31 ± 0.02	4.23 ± 0.02	4.75 ± 0.08	4.76 ± 0.08
	55–59	3.63 ± 0.10	3.35 ± 0.18	3.91 ± 0.03	3.76 ± 0.03	4.22 ± 0.03	4.15 ± 0.03	4.64 ± 0.07	4.67 ± 0.09
	60–64	3.56 ± 0.10	3.29 ± 0.15	3.82 ± 0.03	3.67 ± 0.03	4.14 ± 0.03	4.06 ± 0.03	4.55 ± 0.07	4.57 ± 0.08
	65–70	3.48 ± 0.08	3.19 ± 0.16	3.74 ± 0.03	3.58 ± 0.03	4.04 ± 0.03	3.97 ± 0.03	4.44 ± 0.07	4.48 ± 0.09
TMT-A	50–54	73.20 ± 5.61	86.91 ± 8.02	63.65 ± 1.79	71.67 ± 1.58	58.69 ± 1.51	60.69 ± 1.50	51.49 ± 1.99	47.32 ± 2.34
	55–59	76.71 ± 4.03	92.67 ± 9.07	69.24 ± 1.18	76.81 ± 1.18	63.34 ± 1.26	65.24 ± 1.23	57.11 ± 1.58	52.26 ± 2.40
	60–64	79.22 ± 3.63	93.31 ± 7.67	72.48 ± 0.69	79.99 ± 0.69	66.51 ± 0.73	68.55 ± 0.70	60.23 ± 1.18	55.86 ± 1.82
	65–70	80.25 ± 2.98	96.41 ± 8.30	73.80 ± 0.18	81.52 ± 0.17	68.18 ± 0.16	70.20 ± 0.18	62.06 ± 0.87	57.45 ± 1.93
TMT-B	50–54	185.28 ± 13.15	208.77 ± 19.14	162.40 ± 1.37	172.83 ± 2.43	149.42 ± 1.17	146.31 ± 2.32	133.17 ± 2.74	114.61 ± 4.93
	55–59	185.13 ± 9.54	220.24 ± 21.71	167.50 ± 1.31	182.26 ± 2.61	153.73 ± 1.38	154.64 ± 2.68	138.67 ± 2.64	123.53 ± 5.62
	60–64	188.41 ± 8.62	225.69 ± 18.55	172.22 ± 1.37	191.23 ± 2.68	158.10 ± 1.39	163.61 ± 2.70	143.05 ± 2.63	133.24 ± 4.67
	65–70	191.91 ± 7.39	236.12 ± 19.69	176.56 ± 1.69	200.48 ± 2.97	162.97 ± 1.47	172.66 ± 2.85	148.55 ± 2.74	143.10 ± 5.74
LMT-IR	50–54	8.26 ± 0.71	7.28 ± 0.97	9.99 ± 0.00	9.40 ± 0.00	11.56 ± 0.00	11.35 ± 0.00	13.83 ± 0.35	13.64 ± 0.00
	55–59	8.56 ± 0.52	7.46 ± 0.79	9.99 ± 0.00	9.40 ± 0.00	11.56 ± 0.00	11.35 ± 0.00	13.73 ± 0.34	14.03 ± 0.42
	60–64	8.61 ± 0.45	7.34 ± 0.89	9.99 ± 0.00	9.40 ± 0.00	11.56 ± 0.00	11.35 ± 0.00	13.70 ± 0.34	14.04 ± 0.42
	65–70	8.66 ± 0.38	7.50 ± 0.75	9.99 ± 0.00	9.40 ± 0.00	11.56 ± 0.00	11.35 ± 0.00	13.64 ± 0.31	13.96 ± 0.41
SCWT-IT	50–54	35.18 ± 3.74	30.32 ± 3.10	38.31 ± 0.41	33.42 ± 0.32	36.47 ± 0.45	33.24 ± 0.41	31.26 ± 1.08	30.20 ± 0.74
	55–59	37.66 ± 3.11	30.43 ± 3.79	39.89 ± 0.41	34.69 ± 0.36	38.16 ± 0.56	34.73 ± 0.49	33.65 ± 1.06	32.10 ± 0.88
	60–64	38.78 ± 3.00	31.78 ± 3.45	41.40 ± 0.45	35.94 ± 0.38	39.96 ± 0.59	36.41 ± 0.52	35.67 ± 1.07	34.19 ± 0.74
	65–70	39.97 ± 2.80	32.13 ± 4.07	42.85 ± 0.57	37.28 ± 0.44	42.07 ± 0.66	38.19 ± 0.58	38.37 ± 1.19	36.58 ± 1.00

Predictors in final multivariate linear regression analysis were age, gender and level of education

*AVLT-IR* Auditory Verbal Learning Test—immediate recall, *AVLT-LR* Auditory Verbal Learning Test—long recall, *AVLT-SR* Auditory Verbal Learning Test—short recall, *DSB* digit span backwards, *DSF* digit span forwards, *MMSE* Mini-Mental State Examination, *MoCA* Montreal Cognitive Assessment, *SCWT-IT* Stroop Color-Word Test Interference Trial, *SDMT* Symbol Digit Modalities Test, *TMT* Trail Making Test, *LMT-IR* Logical Memory Test—immediate recall

**Table 10:** Age-, gender-, and education-specific reference values for cognitive tests

		Education level							
		Elementary school		Junior middle school		Senior middle school		College and above	
	Age	Men	Women	Men	Women	Men	Women	Men	Women
MMSE	50–54	24	24	25	25	25	25	26	26
	55–59	24	24	25	25	25	25	26	26
	60–64	24	24	25	25	25	25	26	26
	65–70	24	24	25	25	25	25	26	26
MoCA	50–54	18	18	20	20	21	21	22	23
	55–59	18	17	20	19	21	21	22	22
	60–64	18	17	20	19	21	20	22	22
	65–70	18	17	20	19	21	20	22	22
AVLT-IR	50–54	6	7	8	8	9	10	10	12
	55–59	6	6	7	8	8	10	10	11
	60–64	5	6	7	8	8	9	9	11
	65–70	5	5	6	7	8	9	9	11
AVLT-SR	50–54	1	1	1	2	2	3	3	4
	55–59	1	1	1	2	2	2	2	3
	60–64	0	1	1	1	1	2	2	3
	65–70	0	0	1	1	1	2	2	3
AVLT-LR	50–54	0	0	0	1	1	2	2	3
	55–59	0	0	0	1	1	1	2	2
	60–64	0	0	0	0	0	1	1	2
	65–70	0	0	0	0	0	1	1	2
SDMT	50–54	13	12	19	19	22	25	26	31
	55–59	12	10	17	18	20	23	24	30
	60–64	11	9	15	16	19	22	23	28
	65–70	10	7	14	15	17	20	21	26
DSF	50–54	5	5	6	6	6	6	6	6
	55–59	5	5	5	5	6	6	6	6
	60–64	5	5	5	5	6	6	6	6
	65–70	5	5	5	5	6	6	6	6
DSB	50–54	2	2	2	2	2	2	3	3
	55–59	2	2	2	2	2	2	3	3
	60–64	2	1	2	2	2	2	3	3
	65–70	2	1	2	2	2	2	3	3
TMT-A	50–54	110	123	100	108	95	97	88	84
	55–59	113	129	106	113	100	102	93	89
	60–64	116	130	109	116	103	105	97	92
	65–70	117	133	110	118	105	107	98	94
TMT-B	50–54	281	305	258	269	245	242	229	210
	55–59	281	316	263	278	250	251	235	219
	60–64	284	321	268	287	254	259	239	229
	65–70	288	332	213	296	259	269	244	239
LMT-IR	50–54	1	0	3	2	5	4	7	7
	55–59	2	0.5	3	2	5	4	7	7
	60–64	2	0	3	2	5	4	7	7
	65–70	2	0.5	3	2	5	4	7	7
SCWT-IT	50–54	78	67	86	75	82	74	69	67
	55–59	83	67	89	78	85	78	75	72
	60–64	86	70	92	80	89	81	79	76
	65–70	89	71	96	83	94	85	86	82

Age-, gender-, and education-specific reference values were defined as 1.5 times root mean square error (RMSE) under the mean of normative score for MMSE, MoCA, AVLT-IR, AVLT-SR, AVLT-LR, SDMT, DSF, DSB, and LMT-IR, and 1.5 times RMSE above the mean of normative score for TMT-A, TMT-B, and SCWT-IT

*AVLT-IR* Auditory Verbal Learning Test—immediate recall, *AVLT-LR* Auditory Verbal Learning Test—long recall, *AVLT-SR* Auditory Verbal Learning Test—short recall, *DSB* digit span backwards, *DSF* digit span forwards, *MMSE* Mini-Mental State Examination, *MoCA* Montreal Cognitive Assessment, *SCWT-IT* Stroop Color-Word Test Interference Trial, *SDMT* Symbol Digit Modalities Test, *TMT* Trail Making Test, *LMT-IR* Logical Memory Test—immediate recall

## Discussion

This large community-based study in three Chinese areas is among the first to: 1) examine gender-specific cognitive patterns; 2) explore the gender-specific risk and protective factors; and 3) establish age-, gender-, and education-specific normative data for 12 cognitive tests among a Chinese middle-aged and elderly population. Prior studies mostly employed single or limited cognitive measures and smaller samples to establish restricted normative data [27–29]. Consequently, they may not capture the wide range of cognitive function needed to reflect early changes in mid-life with gender-specific initial ability levels. Thus, encompassing and comparing a wide spectrum of cognitive function may be particularly valuable in identifying modifiable risk factors and critical periods of cognitive impairment following mid-life.

### Gender-specific cognitive patterns

An increasing number of studies carried out in Chinese populations have shown gender-specific cognitive patterns both in China and abroad [30–33]. The rate of global cognitive decline was faster among females than males according to MMSE [30]. In agreement with the Rotterdam Study [34], our study also did not find a rapid change in MMSE score until the age of 70 years which suggests an increased need to pay more attention to a wider range of cognitive domains since the global cognition may be stable before the age of 70 years.

Significant gender disparities were observed in three cognitive domains across different age and education groups. With respect to verbal memory, our results were partially congruent with a growing literature that suggest women perform better than men [35–38]. Interestingly, it has been reported that a female advantage in verbal memory remains consistent throughout the lifespan. Furthermore, a 10-year cohort study found that women outperformed men not only on verbal memory, but also on verbal recognition and semantic fluency tasks [39], suggesting that the female advantage for verbal memory tasks is possibly because women are inclined to use semantic clustering in recall. Contrary to verbal memory, men tended to score higher than women for attention/processing speed/executive function, which is an important cognitive capacity to attend to or to “stay on” a task [40] to complete a task quickly and accurately under the cognitive control of behavior. However, the results only showed the male advantage in the 50–54 and 65–70 years age groups, consistent with previous reports that age-related associations for processing speed were stronger than other domains [41]. The SCWT-IT was interpreted to reflect cognitive flexibility. Van der Elst et al. [42] found clear gender differences on the Stroop interference scores. Nevertheless, the results of regression analyses showed that the influence of age, gender, and education was less profound, which indicated that deficits in Stroop tests may be influenced by intricate factors with concurrent effects.

### Gender-specific risk factors for cognitive performance

Studying gender differences in cognitive function is a complex and controversial topic. Furthermore, the relevance of biological and environmental factors is not yet clear. Given the gaps in our knowledge of the gender-specific associations between these factors and cognition in previous studies, our results may be of special importance.

The effects of education on women were more substantial than in men for general cognition and attention/processing speed/executive function. As we can see from Fig. 3, education could reverse the inferiority in women and even lead to superiority in performance of global and domain-specific cognitive performance. Education may explain most of the gender disparity in cognitive pattern, which was also indicated by Lei et al. from China [31] and Lee et al. from India [43]. With respect to verbal memory, we may presume that education could strengthen the semantic clustering in recall. For attention/processing speed/executive function, the Chinese have a larger male advantage in this domain than Americans, with a potential reason being the relatively equivalent access to formal education in developed countries [40]. In former low-income environments, such as traditional rural China, families may favor sons and large gender gaps in schooling exist in low-income settings. Such long-term educational attainment disparities that Chinese women experience through their life course may affect their cognitive trajectory.

Asides from education, a large range of potentially reversible risk factors for cognitive performance were identified and show gender differences, notably white-collar work, a higher income level, smoking, diabetes, and coronary heart disease for men, and underweight and obesity as well as hypertension for women. Although no significant between-gender differences were observed, the subgroup analysis also indicated that these risk factors should be taken into consideration in the development of gender-specific preventive intervention programs for cognition.

### The need for normative data and a comparison with normative scores

Finally in this study, we provided demographically adjusted and regression-based normative data for 12 cognitive tests. The overall sample size in our study was large and excluded cognitive disorders. The normative data and reference values are finely stratified by the most relevant demographic factors. A quick, efficient, and straightforward method to obtain *z* scores and percentile rank estimates for specific participants is also provided for clinical researchers.

Normative data have been shown to be indispensable for distinguishing normal aging from early transition to cognitive impairment. Undoubtedly, it would be better to endorse age-, gender-, or education-specific cut-off scores based on demographically adjusted normative data in research. As a result, researchers have tried to yield better screening accuracy instead of uniform cut-off scores [44, 45]. Differences are noted when compared with prior studies for normative scores in the Chinese [46, 47]. These differences are likely attributed to distinction in reporting of the normative data. The present study employed a regression-based approach instead of typical methods (e.g., means and SDs calculated from raw scores). The problem intrinsically related to the latter is the need for a relatively smaller size of subgroups [48]. In the regression-based approach, norms are derived from equations by using the data for all the samples and the abovementioned problem disappears with no need for a subdivided sample. Also, the unbalanced data will not affect the norms in the regression-based approach because the estimation of the regression weights cannot be biased by any imbalance in the sample but only results in some loss of statistical power [49]. Furthermore, normative data and an estimated *z* score (and ultimately percentile rank) can even be obtained for particular participants with certain demographic characteristics out of the sample [50].

Certain limitations of this study are noted. First, the present cross-sectional study reported “conventional” norms based on exclusion of participants with evident clinical neurodegenerative diseases instead of “robust” norms that follow individuals longitudinally. It further excludes individuals with subclinical/latent neurological diseases, which may provide less appropriate norms and decreased sensitivity to mild deficits [51], although some research has suggested similarities between two norms in identifying early cognitive impairment [52]. Second, the present study did not take the residential area into consideration, such as a differentiation between urban and rural regions, which may contribute to local differences in education, occupational experiences, income, and lifestyle over the lifespan. Third, since all the medical variables were self-reported, participants may underestimate their symptoms or hesitate to report their real medical status to avoid being perceived as complainers.

## Conclusions

In summary, this study holds significance as it contributes to the ongoing investigation of gender-specific cognitive patterns and predictors of cognitive performance among middle-aged and elderly Chinese. Males were inclined to outperform females in global cognition and attention/processing speed/executive function, while females tended to do better on verbal memory as well as cognitive flexibility. These cognitive disparities were considerably mitigated or even reversed but not fully explained by education. Meanwhile, the regression-based and demographically adjusted normative score was provided for 12 cognitive tests to serve as an additional resource and guidance for clinical researchers. Taken together, our findings call for future longitudinal follow-up to improve our knowledge of cognitive patterns and related risk factors. We believe that better understanding the biology of gender differences in cognitive patterns will not only be conducive to advocating a healthy lifestyle and promoting gender-specific interventions to prevent or minimize cognitive impairment but will also be integral to the investigation of personalized, gender-specific new therapies.

## Additional file

Additional file 1: Supplementary methods and results. (DOCX 34 kb)

## Abbreviations

AD: Alzheimer’s disease
ADI: Alzheimer’s Disease International
AVLT: Auditory Verbal Learning Test
AVLT-IR: Auditory Verbal Learning Test—immediate recall
AVLT-LR: Auditory Verbal Learning Test—long recall
AVLT-SR: Auditory Verbal Learning Test—short recall
BMI: Body mass index
DSB: Digit span backwards
DSF: Digit span forwards
EMCOA: Effects and Mechanism of Cholesterol and Oxysterol on Alzheimer’s disease
FDR: False discovery rate
LMT-IR: Logical Memory Test—immediate recall
MCI: Mild cognitive impairment
MMSE: Mini-Mental State Examination
MoCA: Montreal Cognitive Assessment Test
PCA: Principal component analysis
SCWT-IT: Stroop Color-Word Test Interference Trial
SD: Standard deviation
SDMT: Symbol Digit Modalities Test
TMT: Trail Making Test
WMS-RC: Wechsler Memory Scale Revised for China
