# Supplementary material for: Patterns of cognitive function in middle-aged and elderly Chinese adults—findings from the EMCOA study
Source: Alzheimers Res Ther. 2018 Sep 15;10:93. doi: 10.1186/s13195-018-0421-8 (PMC6138914; doi:10.1186/s13195-018-0421-8)
Supplement: Supplementary file 1 — Supplementary methods and results. (DOCX 34 kb) [file 13195_2018_421_MOESM1_ESM.docx]

**Supplementary methods and results**

***2.3 Cognitive test battery***

***Mini-Mental State Examination***^[1]^ Mini-mental state examination (MMSE) is the most widely used multi-domain cognitive screening test that comprises 20 subtests and totaling 30 points. It offers broad coverage of cognitive domains for orientation, attention and calculation, language, word recall and visuo-construction. However, problems of ceiling effects and limited dynamic performance with MMSE have been well-described, which can be attributed to the absence of executive function items as well as a lack of complexity.

***Montreal Cognitive Assessment Test***^[2]^ Montreal Cognitive Assessment Test (MoCA) was developed as the research increasingly focuses on pre-stage of dementia and the growing need for earlier diagnosis and management. As another multi-domain cognitive screening instrument scored out of 30 points, not only does MoCA offer many of the same advantages of the MMSE, it also involves executive function, higher-level language abilities and more complex visuospatial processing, which makes it more challenging with less ceiling effect.

***Auditory verbal learning test***^[3]^ Auditory verbal learning test (AVLT) is a 3-trial, 12-item word list learning test with a score range of 0-12. It provides information on the immediate and delayed verbal recall as well as the ability of memory storage and retrieval. A list of 12 words belonging to three semantic categories is read by interviewers to the participants over 3 trials. After each trial, the participants were required to recall as many words as possible in any desired order. Immediate verbal recall was measured by the sum number of words for all three attempts. Short and long recall (AVLT-SR & AVLT-LR) are defined as the number of words freely recalled respectively from a short (5min) delay recall trial and a long (20 min) delay recall trial following distraction task. Each correctly recalled word was recorded as one point and higher scores indicate better performance.

***Symbol Digit Modalities Test*** ^[4]^Symbol digit modalities test (SDMT) is a rapid simple substitution task to assess attention, executive function and processing speed, in which a reference key is given to pair specific numbers with given geometric figures as quickly as possible. The score is the total number of correct substitutions within 90-seconds and the range of possible scores is 0 to 100 with higher scores indicating better performance.

***Logical Memory Test-Immediate Recall***^[5]^ **Logical memory test-immediate recall (LMT-IR)** is a test of immediate verbal memory from the Wechsler Memory Scale-Revised of China (WMS-RC). In this test, two logically related stories are verbally and separately presented to the participants and contain a total of 19 and 30 units of information respectively. The interviewers read the stories out loud one by one to the participants who then attempt to restate the story immediately. The 49 units of information are gist scored (range 0–24.5) with higher scores indicating better memory.

***Digit span forwards and backwards***^[6]^ Digit span forwards & backwards (DSF & DSB) tests are subtests of Wechsler Adult Intelligence Scale-Revised of China (WAIS-RC). Attention, immediate recall and working memory were assessed. The participants were required to repeat a string of numbers respectively in a forward or backward order. The number of repeated digits increase by 1 until the participants fail two consecutive trials for same digit span. The total score was recorded as the number of the longest series repeated correctly with a maximum score of 11 for DSF and 9 for DSB.

***Trail Making Test- A&B***^[7]^ Trail making test-A&B (TMT-A&B) consists of two separate procedures that are administered to evaluate processing speed and cognitive flexibility. For TMT-A, a test of processing speed, the participants are required to connect numbered circles randomly scattered across a page in sequential order from 1 to 25 as quickly as possible. TMT-B is a Chinese modified test of processing speed and cognitive flexibility in which participants are required to connect the 25 numbers enclosed in 13 squares and 12 circles in ascending order alternating between two shapes that are randomly scattered on a page. The score is the time in seconds needed for completion of the tests with a maximum allotted time of 150 seconds for TMT-A and 360 seconds for TMT-B. A lower score indicates better performance.

***Stroop Color-Word Test-Interference Trial***^[8]^ **The** **Stroop color-word test-interference trial (SCWT-IT)** is used to measure the ability to resist interference and assess selective attention, cognitive flexibility, response inhibition and executive function. It consisted of three subtests: reading words denoting four different colors printed in black (subtest A), reading color of dots printed in denoted color (subtest B), and reading the [incongruent](javascript:void(0)) color of printed words (subtest C) as quickly as possible. The Stroop interference effect refers to the inhibition of an over-learned response (read words printed in colors) by a competing response (name the colors of the printed words). The interference score is calculated by subtracting the time in seconds needed for subtest B from subtest C with a higher interference score corresponding to worse performance.

***2.4 Covariates***

Educational level was categorized according to the Chinese education system and divided into four groups: Elementary school (≤6 years of education), Junior middle school (7–9 years of education), Senior middle school (10-12 years of education) and College and above (at least college or university; ≥13 years of education). Three categories were defined according to the participants’ monthly income indicating a low (≤1500 China Yuan (CNY)), medium (1500-3000 CNY) or high (≥3000 CNY). Occupation was grouped into two main categories of white-collar work and manual work^[9]^. Weight and height were measured to calculate body mass index (BMI) as the ratio of weight to squared height (kg/m^2^). It was further used to define underweight (<18.5 kg/m^2^), normal weight (18.5-23.9 kg/m^2^), overweight (24-27.9 kg/m^2^), and obesity (≥28 kg/m^2^) according to exclusive BMI cut-offs for Chinese people put forward by the Working Group on Obesity in China (WGOC) in 2003^[10]^. Residential status was categorized as [solitude](file:///D:\Youdao\Dict\7.5.0.0\resultui\dict\?keyword=solitude) or not. Physical activity was dichotomized as physically active if they were active during exercise including walking, bicycling, tai Chi martial arts and yoga or other similar activities more than 30 min at least 3 days in a week, or physically inactive if they were active less than that^[11]^. Participants were asked whether or not and how often they undertook activities of reading newspapers, books and magazines. Responses for an approximate number of days per week were identified as having reading habits and responses for not reading at all, once a year or less or several times a year were identified as not. Smoking and drinking status was determined by self-report and dichotomized as current smoker/drinker or not. Participants were classified as current smokers if the number of cigarettes they smoked per week was greater than zero more than six months before enrollment. Current drinker is defined as drink alcohol for 50 ml or more at least once per week more than six months before enrollment. Relevant past and family medical data, including physician diagnosed diabetes, hypertension, stroke, coronary heart disease, were requested from participants, family, or hospitals for examination and validation.

***2.5 Data analysis of the development of normative data for 12 cognitive tests and related Z-score***

Results from hierarchical multiple regression models in Table 7 provided the proportion of variance accounted for age, gender and education and other factors together in cognitive performance with enter method (including all of the variables regardless of the statistical significance in one step). The increment in R^2^ associated with the inclusion of other factors is also provided. The hierarchical multivariate linear regression analyses showed that age, gender and years of education accounted for the largest portions of the variance in all the cognitive tests. These results indicate that researchers and clinicians need to take age, gender and education into consideration for an accurate interpretation of the different cognitive tests. The expected “normal” test score can be estimated based on age, gender and education from multivariate regression models. These demographic characteristics from a given individual can be weighted by the corresponding parameter estimates and then summed to provide the expected score for that individual, which makes it possible to determine whether the actual score of an individual is within expected normal limits. Therefore, in order to determine a middle-aged and elderly Chinese’s “expected” performance, another stepwise multiple linear regression analyses (including statistically significant variables in the regression in final step) were then performed to predict expected 12 cognitive tests with age, gender and education as predictors. The predictive models contained age and education years as continuous variables and coded gender as 1 for male and 2 for female. Interactions between every two predictors and nonlinear associations using quadratic terms for education years and age were tested. The final model only retained variables that significantly contributed to prediction of score. The unstandardized beta weights of the predictors in the final predictive model together with the constant were used to calculate the predicted scores on each test and develop the normative data.

For each cognitive test, we created a multiple regression equation specific to age, gender and education and their interactions and nonlinear associations, which was aimed to first predict the theoretical cognitive score for an individual with the same age (years), education (years), and gender (coded as 1=male, 2=female). The predictive regression equations with root mean square error (RMSE) for each cognitive test were as follows:

1. MMSE_predicted_ =0.084*Education+0.078*Education*Gender-1.041*Gender+27.599 (RMSE: 1.999);
2. MoCA_predicted_=0.233*Education-0.031*Age*Gender+0.127*Education*Gender+23.136 (RMSE: 3.138);
3. AVLT-IR_predicted_=0.282*Education+0.110*Education*Gender-0.001*Age^2^+12.727 (RMSE: 4.67446);
4. AVLT-SR_predicted_ = 0.060*Education*Gender-0.001*Age^2^+0.006*Education^2^+5.028 (RMSE: 2.376);
5. AVLT-LR_predicted_ = 0.007*Education^2^+0.057*Education*Gender-0.001*Age^2^+4.505 (RMSE: 2.609);
6. SDMT_predicted_=0.461*Education-0.323*Age+0.681*Education*Gender-5.477*Gender+45.410 (RMSE: 9.829);
7. DSF_predicted_=0.186*Education-0.001*Age*Education+6.502 (RMSE: 1.381);
8. DSB_predicted_=0.004*Education^2^-0.019*Age-0.391*Gender+0.026*Education*Gender+4.864 (RMSE: 1.217);
9. TMT-A_predicted_ = 5.309*Age-1.877*Education*Gender-0.039Age^2^+24.601*Gender-114.999 (RMSE: 24.256);
10. TMT-B_predicted_ = 0.969*Age*Gender-4.493*Education*Gender+152.416 (RMSE: 63.911);
11. LMT-IR_predicted_ = 0.019*Education^2^-1.751*Gender+0.129*Education*Gender+9.071 (RMSE: 4.66284);
12. SCWT-IT_predicted_ = -0.277*Gender-0.004*Education^2^+0.001*Age*Education+0.015*Gender*Education+3.686 (RMSE: 0.534).

The predictive scores can then be used to generate demographically-adjusted Z-scores and percentiles by subtracting the predicted test score from the observed test score and dividing by the standard error of the estimate. The resultant Z-score can be converted to a percentile that indicates the individual’s cognitive performance among peers of comparable age, gender, and education^[12]^. The following formula was used to calculate Z-scores in our models:

Z-score = $\frac{Raw Score-Predicted Score}{RMSE}$

where:

Z-score is the estimate for an individual subject;

Raw score is the score the individual subject obtained from a given test;

Predicted score is from multivariate regression model for given population;

RMSE is the root mean square error of the regression equation.

For illustrative purposes, if there is interest in predicting theoretical MoCA score for a population of 60-year-old women with 9 years of education, we enter these variables into the above equation of predicted MoCA score (that is, Age = 60, Education=9, Gender=2) to obtain the predicted score of 23.80. Next, if we would like to obtain the Z-score (and ultimately percentile rank) for the MoCA score of a particular 60-year-old woman with 9 years of education who scores a 24 on the MoCA, then we can use the above formula to obtain the Z-score of 0.06 (the RMSE is 3.14). The value corresponds to a percentile of 49.90, and we have thus obtained one estimate of the individual’s MoCA score as approximately at the 50th percentile.

**Reference**

[1] Folstein M F, Folstein S E, Mchugh P R: "Mini-mental state". A practical method for grading the cognitive state of patients for the clinician, J Psychiatr Res, 1975, 12(3): 189-198.

[2] Nasreddine Z S, Phillips N A, Bedirian V, et al: The Montreal Cognitive Assessment, MoCA: a brief screening tool for mild cognitive impairment, J Am Geriatr Soc, 2005, 53(4): 695-699.

[3] Ma J, Zhang Y, Guo Q: Comparison of vascular cognitive impairment--no dementia by multiple classification methods, Int J Neurosci, 2015, 125(11): 823-830.

[4] Price K L, Desantis S M, Simpson A N, et al: The impact of clinical and demographic variables on cognitive performance in methamphetamine-dependent individuals in rural South Carolina, Am J Addict, 2011, 20(5): 447-455.

[5] Wang C, An Y, Yu H, et al: Association between Exposure to the Chinese Famine in Different Stages of Early Life and Decline in Cognitive Functioning in Adulthood, Front Behav Neurosci, 2016, 10: 146.

[6] Miu J, Negin J, Salinas-Rodriguez A, et al: Factors associated with cognitive function in older adults in Mexico, Glob Health Action, 2016, 9: 30747.

[7] Wei M, Shi J, Li T, et al: Diagnostic Accuracy of the Chinese Version of the Trail-Making Test for Screening Cognitive Impairment, J Am Geriatr Soc, 2017.

[8] Chan R C, Hoosain R, Lee T M, et al: Are there sub-types of attentional deficits in patients with persisting post-concussive symptoms? A cluster analytical study, Brain Inj, 2003, 17(2): 131-148.

[9] Soltysik B K, Kroc L, Piglowska M, et al: An Evaluation of the Work and Life Conditions and the Quality of Life in 60 to 65 Year-Old White-Collar Employees, Manual Workers, and Unemployed Controls, J Occup Environ Med, 2017, 59(5): 461-466.

[10] Chen C, Lu F C: The guidelines for prevention and control of overweight and obesity in Chinese adults, Biomed Environ Sci, 2004, 17 Suppl: 1-36.

[11] Frederiksen K S, Verdelho A, Madureira S, et al: Physical activity in the elderly is associated with improved executive function and processing speed: the LADIS Study, Int J Geriatr Psychiatry, 2015, 30(7): 744-750.

[12] Shirk S D, Mitchell M B, Shaughnessy L W, et al: A web-based normative calculator for the uniform data set (UDS) neuropsychological test battery, Alzheimers Res Ther, 2011, 3(6): 32.
